# Specific Xylan Activity Revealed for AA9 Lytic Polysaccharide Monooxygenases of the Thermophilic Fungus *Malbranchea cinnamomea* by Functional Characterization

**DOI:** 10.1128/AEM.01408-19

**Published:** 2019-11-14

**Authors:** Silvia Hüttner, Anikó Várnai, Dejan M. Petrović, Cao Xuan Bach, Dang Thi Kim Anh, Vu Nguyen Thanh, Vincent G. H. Eijsink, Johan Larsbrink, Lisbeth Olsson

**Affiliations:** a Department of Biology and Biological Engineering, Division of Industrial Biotechnology, Chalmers University of Technology, Gothenburg, Sweden; b Wallenberg Wood Science Center, Chalmers University of Technology, Gothenburg, Sweden; c Faculty of Chemistry, Biotechnology and Food Science, Norwegian University of Life Sciences (NMBU), Ås, Norway; d Centre for Industrial Microbiology, Food Industries Research Institute, Hanoi, Vietnam; University of Toronto

**Keywords:** biomass degradation, hemicellulose, monooxygenases, thermophilic

## Abstract

The thermophilic biomass-degrader Malbranchea cinnamomea exhibits poor growth on cellulose but excellent growth on hemicelluloses as the sole carbon source. This is surprising considering that its genome encodes eight lytic polysaccharide monooxygenases (LPMOs) from auxiliary activity family 9 (AA9), enzymes known for their high potential in accelerating cellulose depolymerization. We characterized four of the eight (*M. cinnamomea* AA9s) *Mc*AA9s, namely, *Mc*AA9A, *Mc*AA9B, *Mc*AA9F, and *Mc*AA9H, to gain a deeper understanding about their roles in the fungus. The characterized *Mc*AA9s were active on hemicelluloses, including xylan, glucomannan, and xyloglucan, and furthermore, in accordance with transcriptomics data, differed in substrate specificity. Of the *Mc*AA9s, *Mc*AA9H is unique, as it preferentially cleaves residual xylan in phosphoric acid-swollen cellulose (PASC). Moreover, when exposed to cellulose-xylan blends, *Mc*AA9H shows a preference for xylan and for releasing (oxidized) xylooligosaccharides. The cellulose dependence of the xylan activity suggests that a flat conformation, with rigidity similar to that of cellulose microfibrils, is a prerequisite for productive interaction between xylan and the catalytic surface of the LPMO. *Mc*AA9H showed a similar trend on xyloglucan, underpinning the suggestion that LPMO activity on hemicelluloses strongly depends on the polymers’ physicochemical context and conformation. Our results support the notion that LPMO multiplicity in fungal genomes relates to the large variety of copolymeric polysaccharide arrangements occurring in the plant cell wall.

**IMPORTANCE** The *Malbranchea cinnamomea* LPMOs (*Mc*AA9s) showed activity on a broad range of soluble and insoluble substrates, suggesting their involvement in various steps of biomass degradation besides cellulose decomposition. Our results indicate that the fungal AA9 family is more diverse than originally thought and able to degrade almost any kind of plant cell wall polysaccharide. The discovery of an AA9 that preferentially cleaves xylan enhances our understanding of the physiological roles of LPMOs and enables the use of xylan-specific LPMOs in future applications.

## Abstract (precis)

The *Malbranchea cinnamomea* LPMOs (*Mc*AA9s) showed activity on a broad range of soluble and insoluble substrates, suggesting their involvement in various steps of biomass degradation besides cellulose decomposition. Our results indicate that the fungal AA9 family is more diverse than originally thought and able to degrade almost any kind of plant cell wall polysaccharide. The discovery of an AA9 that preferentially cleaves xylan enhances our understanding of the physiological roles of LPMOs and enables the use of xylan-specific LPMOs in future applications.

## INTRODUCTION

Lytic polysaccharide monooxygenases (LPMOs; EC 1.14.99.53-56) are a recently discovered group of copper enzymes that cleave a broad range of oligo- and polysaccharides oxidatively, using an exogenous source of electrons (1, 2). They are classified as enzymes with Auxiliary Activities (AAs) in the Carbohydrate Active enZymes database (CAZy; http://www.cazy.org) (3) and to date have been divided into seven families: AA9, AA11, and AA16 (fungi), AA10 (bacteria or viruses), and AA15 (animal, oocytes, viruses, or algae) enzymes act on *β*-1,4-linked polysaccharides such as cellulose, chitin, or xyloglucan, while AA13 (fungi) degrades *α*-1,4-linked polymers such as starch and AA14 enzymes (fungi) act solely on xylan (1, 4). LPMOs were originally thought to be monooxygenases, using oxygen as a cosubstrate (2, 5, 6), but recent studies indicate that they act as peroxygenases, using hydrogen peroxide as a cosubstrate (7, 8). The outcome is, in either case, the oxidation of the target polysaccharide at the C_1_ or C_4_ carbon of the scissile glycosidic bond (C_1_ or C_4_ oxidation), resulting in bond destabilization (9). The chain breaks introduced by LPMOs acting on crystalline substrates disrupt the crystalline packing and serve as access points for further degradation by hydrolases, such as cellobiohydrolases or endoglucanases (2, 10–12).

LPMOs play a vital role in the decomposition of plant biomass in nature and have gained importance in industrial processes aimed at the degradation of recalcitrant polysaccharides to fermentable sugars (13–15). In the depolymerization of cellulose, the most abundant polymer in land plants and the primary feedstock in biorefineries, cellulose-active LPMOs act synergistically with hydrolytic enzymes, increasing the saccharification efficiency (16, 50). LPMOs also potentially could boost cellulose degradation by targeting the hemicelluloses, such as xylan, that coat and are strongly bonded to the cellulose surface and thereby restrict access of cellulolytic enzymes (17, 18). However, information on xylan-active LPMOs and their interplay with other enzymes is still scarce. Importantly, Couturier et al. recently described the first two AA14 LPMOs, which seem to target xylan only, likely acting on junction zones of xylan and cellulose (19). Xylan activity has also been described for only three members of the large AA9 family, but in all cases activity on xylan was weak and much lower than the activity on cellulose (20–22). Many questions remain concerning xylan-active LPMOs, such as the structural determinants of their activity on xylan, their physiological relevance, how widespread they are in microorganisms, and the role substrate conformation plays in enzymatic activity.

Since 2010, more than 30 AA9 LPMOs have been reported that act on *β*-1,4-linked plant cell wall polysaccharides, including (insoluble) cellulose as well as (soluble) xyloglucan, glucomannan, and cellooligosaccharides (5, 18, 23). Despite studies on the interaction of LPMOs with polysaccharides, the structural or physiological reasons for the observed substrate promiscuity remain unknown (24, 25). Similarly, the biological role of LPMO multiplicity has not been elucidated. Genomes of fungal biomass degraders encode multiple (on average, seven) AA9 genes, with some species harboring up to 40 (26). As suggested by Horn et al., it is quite feasible that the multiple enzymes from one organism have different substrate specificities, not only in terms of which polymer they cleave but also in terms of the copolymeric context of the polymer in the plant cell wall (27). Since the majority of LPMO studies have concentrated on characterizing a single enzyme, a bigger picture about a species’ need for multiple AA9s, and their respective roles in plant cell wall depolymerization, is lacking.

A recent study of the transcriptome of the compost-dwelling, thermophilic fungus *Malbranchea cinnamomea* revealed several putative AA9 LPMOs that were upregulated during growth on beechwood xylan (28), suggesting an involvement in the utilization of this polymer. The conspicuous lack of growth of *M. cinnamomea* on cellulosic substrates was a further indication that its eight putative AA9 LPMOs have primary functions other than cellulose degradation. Therefore, we set out to characterize *M. cinnamomea* LPMOs, hoping to find novel LPMO activities targeting hemicelluloses. We revealed multiple LPMOs with broad hemicellulolytic activity, including activity on xylan, and report an AA9 LPMO with a clear preference for this abundant polysaccharide. We also show that the various LPMO activities depend on the copolymeric context of the substrate.

## RESULTS

### *M. cinnamomea* encodes eight putative AA9 LPMOs.

A previous transcriptomics study (28) showed that four of the eight putative AA9 genes in *M. cinnamomea* (order *Onygenales*) FCH 10.5, namely, *mclpmo9b*, *mclpmo9e*, *mclpmo9f*, and *mclpmo9h*, were upregulated during growth on wheat bran compared to that on glucose. Furthermore, *mclpmo9f* and *mclpmo9h* were also highly upregulated on beechwood xylan as the sole carbon source (see File S1a in the supplemental material), suggesting involvement in xylan degradation. Since *M. cinnamomea* FCH 10.5 showed poor growth on cellulosic substrates but substantial growth on hemicelluloses like xylan and glucomannan (Fig. S1) (28), we hypothesized that its putative AA9 enzymes could act on hemicelluloses, in particular on xylan. In the present study, we were able to express five of the eight proteins, namely, *Mc*AA9A, *Mc*AA9B, *Mc*AA9C, *Mc*AA9F, and *Mc*AA9H, in Pichia pastoris with their native signal peptides and without C-terminal tags. Analysis of the purified proteins on SDS-PAGE with and without a preceding endoglycosidase H treatment (Fig. S2a) indicated that only *Mc*AA9A was N-glycosylated, which agrees with it having predicted N-glycosylation sites at Asn160 and Asn208. Binding assays with Avicel showed similar weak binding for all five proteins, in accordance with the absence of carbohydrate-binding modules (CBMs) (File S1b). Four of the purified enzymes, *Mc*AA9A, -B, -F, and -H, produced H_2_O_2_ in the presence of ascorbic acid but not when the reducing agent was omitted (Fig. S2b). This oxidase activity is a futile side reaction of LPMOs (29), and the results of the assay suggest that the four H_2_O_2_-producing *Mc*AA9s were correctly folded and functional. No significant H_2_O_2_ production was detected for the fifth purified enzyme, *Mc*AA9C, so it was excluded from further experiments.

### Sequence and structure comparison of *Mc*AA9s.

**FIG 1:**
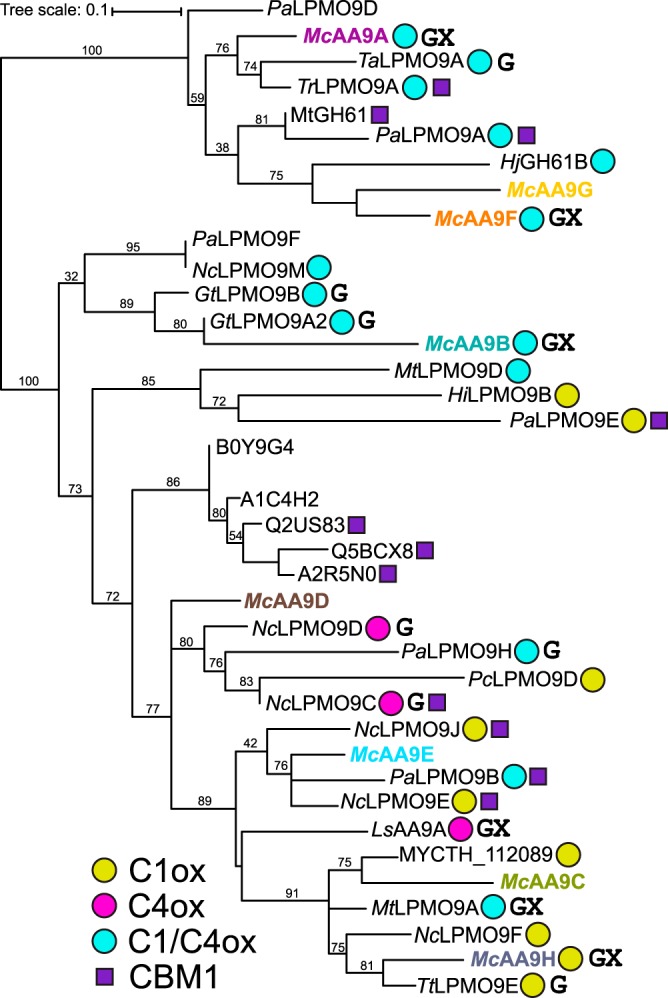
Phylogenetic tree of the catalytic domains of *Mc*AA9s and other AA9 LPMOs. The oxidative regio-specificity of LPMOs (C_1_ox, C_4_ox, and C_1_/C_4_ox) for which activity on cellulose has been demonstrated is indicated by yellow, magenta, and cyan circles and was assigned based on published experimental evidence (for a list of references, see File S1d). Known activities on hemicelluloses are indicated by a G for activity on beta-glucans and X for activity on xylan. Note that these activities may sometimes only be detectable when mixing the hemicellulose with cellulose, as discussed in the text. Also note that not all shown LPMOs have been tested with all substrates. The (predicted) presence of carbohydrate binding module 1 (CBM1) is indicated by a purple square. *Pa*, Podospora anserina; *Mc*, *Malbranchea cinnamomea*; *Ta*, *Thermoascus aurantiacus*; *Tr*, Trichoderma reesei; *Hj*, Hypocrea jecorina (*T. reesei*); *Nc*, Neurospora crassa; *Gt*, Gloeophyllum trabeum; *Mt*, *Myceliophthora thermophila*; *Hi*, Heterobasidion irregulare; *Pc*, Phanerochaete chrysosporium; *Ls*, *Lentinus similis*; *Tt*, Thielavia terrestris. MYCTH_112089 is from *M. thermophila*; B0Y9G4 to A2R5N0 are from *Aspergillus* species. For multiple-sequence alignment, see Fig. S3.

The *Mc*AA9s were found to be quite diverse, with sequence identities of the catalytic AA9 domains ranging from 29% to 61% (File S1c). Phylogenetic analysis of the catalytic domains showed that the *Mc*AA9s do not cluster together but are spread out among other characterized AA9 LPMOs (Fig. 1). *Mc*AA9A, -B, -F, and -G grouped with known C_1_/C_4_-oxidizing LPMOs, while *Mc*AA9C, -D, -E, and -H were found in clades with mixed regio-specificity. All eight proteins appear to be typical secreted LPMOs with predicted AA9 domains comprising 213 to 229 residues and starting with a histidine residue following the N-terminal signal peptide (File S1b). While about 20% of known AA9 LPMOs contain a CBM (27), none of the *Mc*AA9s has a CBM. Only *Mc*AA9A has an additional C-terminal domain that comprises approximately 60 residues and does not show significant similarity with known CBMs or other domains of known function. All *Mc*AA9s have two cysteine residues that are putatively involved in forming a disulfide bond between loop L2 and strand β8 (30). *Mc*AA9D has two additional cysteine residues (Cys152 and Cys234) that might form a second disulfide bond, which is only present in about one-third of the enzymes in the multiple-sequence alignment (MSA) (Fig. S3). Homology models generated with Phyre2 (31) showed for all eight *M. cinnamomea* proteins the typical immunoglobulin G (IgG)-like β-sandwich fold and a histidine brace motif that holds the copper ion in place (reviewed recently in reference 32). Predicted sites for N-glycosylation are all located far away from the flat surface region that interacts with the substrate (Fig. S4).

### *Mc*AA9H preferentially cleaves xylan.

**FIG 2:**
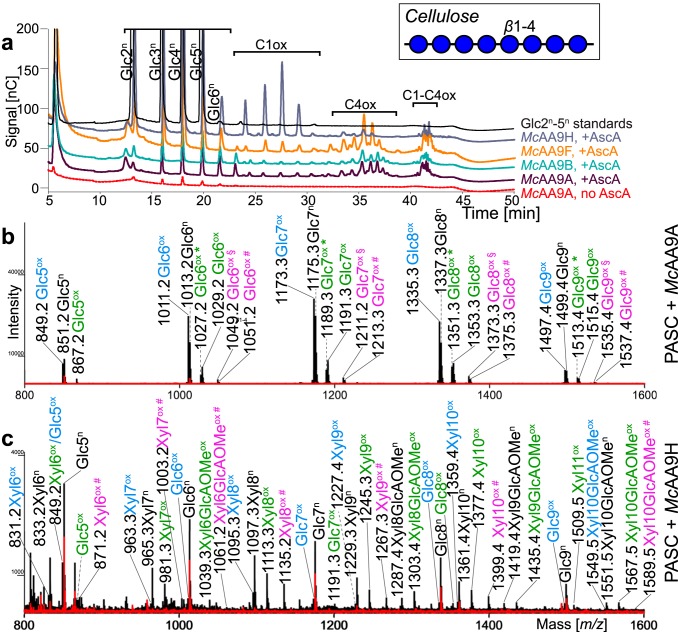
Activity of *Mc*AA9s on cellulose. (a) HPAEC-PAD elution pattern of phosphoric acid swollen cellulose (PASC) after incubation with *Mc*AA9s. Enzymes (1 μM) were incubated in 50 mM BisTris buffer, pH 6.1, and 0.3% (wt/vol) PASC for 4 h at 40°C with (+AscA) or without (no AscA) 1 mM ascorbic acid. Control reactions without ascorbic acid showed that all AA9 preparations had a minor cellulolytic background activity, particularly *Mc*AA9H (Fig. S5). However, for AA9s with a strong C_4_-oxidizing activity, most of the detected native products stem from expected on-column degradation of C_4_-oxidized oligomers (49). Product formation over time is shown in Fig. S5d to f. (b and c) MALDI-TOF MS spectra showing the sodium adducts of reaction products of the same reactions as that shown in panel a containing *Mc*AA9A (b) and *Mc*AA9H (c), with ascorbic acid (black spectra) or without ascorbic acid (red spectra). Labeled peaks show the masses of monosodium adducts of native oligosaccharides (n, in black), C_1_-oxidized lactone, C_4_-oxidized ketoaldose species (Δ*m/z* −2 Da, ox, in blue), hydrated C_1_-oxidized aldonic acid, C_4_-oxidized gemdiol species (Δ*m/z* +16 Da, ox, in green), hydrated double oxidized species (Δ*m/z* +14, ox *, in green), the sodium salt of hydrated double oxidized oligosaccharides (Δ*m/z* +36 Da, ox §, in pink), or the disodium adducts of hydrated C_1_-oxidized products (Δ*m/z* +38 Da, ox #, in pink). Labels for signals corresponding to xylooligosaccharides are shaded in yellow. Note that sodium salts of GlcAOMe-containing compounds (ox #, in pink) do not necessarily prove C_1_ oxidation, since both the aldonic acid and the GlcAOMe can engage in salt formation. Note that oxidized cellooligomers are rare in the mass spectrum shown in panel c; the native cellooligomers in this spectrum likely stem from background activity (Fig. S5). Additional and more detailed MS analyses of products generated by all four LPMOs are provided in Fig. S6.

Since all previously characterized AA9 LPMOs are active on cellulose, the four *Mc*AA9s were first assayed for their ability to cleave phosphoric acid swollen cellulose (PASC) in the presence of ascorbic acid as an electron donor. High-performance anion-exchange chromatography coupled with pulsed amperometric detection (HPAEC-PAD) analysis showed that *Mc*AA9A, -B, and -F produced native as well as C_1_-, C_4_-, and C_1_/C_4_-oxidized cellooligosaccharides from PASC (Fig. 2a and Fig. S5a and b). Products were detectable already after 30 min of incubation, and product formation continued for up to 24 h (Fig. S5d to f). Controls without enzyme showed no product formation (Fig. S5c), while controls without ascorbic acid revealed an endoglucanase background activity that most likely stemmed from native *Pichia* enzymes that had been insufficiently removed in the protein purification process. This background activity resulted in the formation of native, but not oxidized, cellooligosaccharides in reactions without ascorbic acid. Matrix-assisted laser desorption-ionization time-of-flight mass spectrometry (MALDI-TOF MS) further confirmed the presence of native and oxidized products (*Mc*AA9A [Fig. 2b] and *Mc*AA9A, -B, and -F [Fig. S6a to c]). Both HPAEC chromatograms, with large peaks for the C_4_-oxidized products, and the mass spectra, showing small signals for the diagnostic sodium salts formed by C_1_ oxidized products, indicate that these three LPMOs predominantly oxidize cellulose at the C_4_ position.

In contrast to the other three tested *Mc*AA9s, *Mc*AA9H primarily produced C_1_-oxidized cellooligosaccharides from PASC (Fig. 2a and Fig. S5). Intriguingly, MALDI-TOF MS showed not only signals for cellooligosaccharides but also peaks of similar or even higher intensity corresponding to native and oxidized xylooligosaccharides, with or without methylglucuronic acid (GlcAOMe) side groups (Fig. 2c and Fig. S6d). The likely sources of these xylan fragments are traces of xylan remaining in commercial microcrystalline cellulose, i.e., Avicel PH-101, that was used for PASC preparation. In fact, Avicel contains about 1 to 2% xylan, which has been shown to restrict cellulose accessibility by cellulases (33, 34). Considering the low level and low accessibility of xylan in PASC, the high relative abundance and variety of xylan-derived products liberated by *Mc*AA9H indicate a preference for structural xylan. The product spectra showed relatively strong signals for the nonhydrated oxidized (Δ*m/z* –2) and hydrated oxidized (Δ*m/z* +16) xylan fragments, indicating the formation of aldonic acid or gemdiol species, resulting from C_1_ or C_4_ oxidation. The presence of sodium salts of the aldonic acids of non-GlcAOMe-containing xylooligosaccharides (Δ*m/z* +38) confirmed the occurrence of C_1_ oxidation, but the data did not allow conclusions about the ratio between C_1_ and C_4_ oxidation of xylan.

### Coating on cellulose changes the susceptibility of hemicelluloses to degradation by *Mc*AA9s.

**FIG 3:**
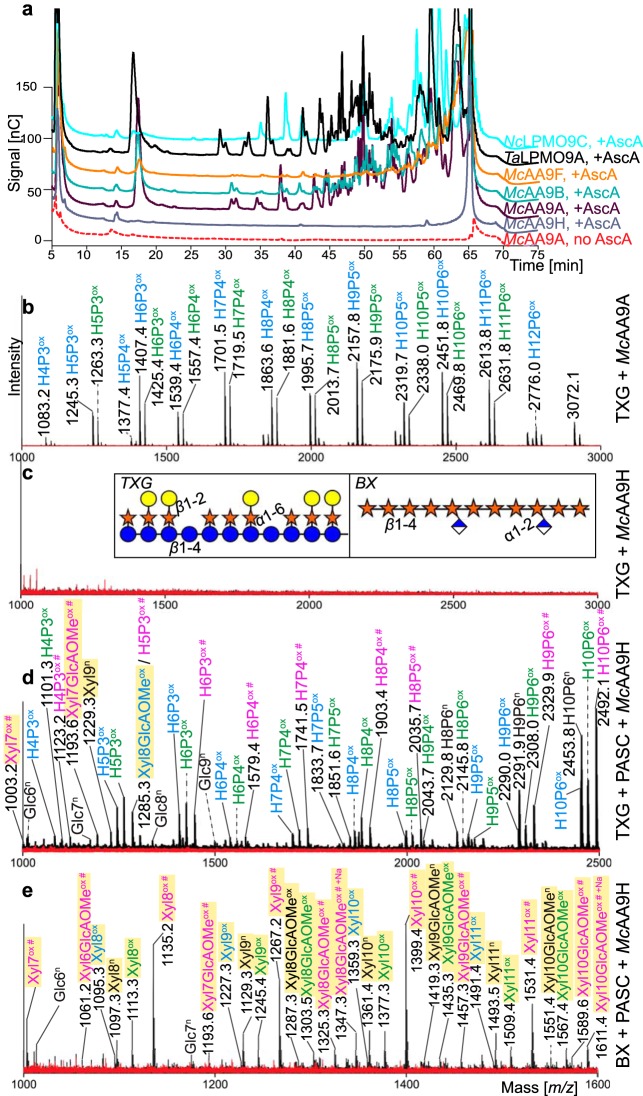
Activity of *Mc*AA9s on tamarind xyloglucan (TXG) and TXG- and birchwood xylan (BX)-coated cellulose. (a) HPAEC-PAD chromatograms of products generated from TXG after incubation with *Mc*AA9s and two LPMOs known to be active on TXG, *Nc*LPMO9C and *Ta*LPMO9A. Enzymes (1 μM) were incubated in 50 mM BisTris buffer, pH 6.1, containing 0.3% (wt/vol) TXG for 3.5 h at 40°C with (+AscA) or without (no AscA) 1 mM ascorbic acid (see Fig. S7 for product formation over time and additional control reactions without AscA). (b and c) MALDI-TOF MS spectra highlighting the sodium adducts of reaction products generated by *Mc*AA9A (b) and *Mc*AA9H (c), in the presence (black spectra) or absence of ascorbic acid (red spectra). MS data for the other *Mc*AA9s, *Nc*LPMO9C and *Ta*LPMO9A, are shown in Fig. S7. The insets in panel c depict the structures of TXG and BX: glucose (blue circles), xylose (orange stars), galactose (yellow circles), and GlcAOMe (blue-white diamonds). (d and e) MALDI-TOF MS spectra highlighting the sodium adducts of reaction products generated upon incubation of *Mc*AA9H with TXG + PASC (d) or BX + PASC (e). Labeled peaks show the masses of monosodium adducts of native oligosaccharides (n, in black), C_1_-oxidized lactone or C_4_-oxidized ketoaldose species (Δ*m/z* −2 Da, ox, in blue), hydrated C_1_-oxidized aldonic acid or C_4_-oxidized gemdiol species (Δ*m/z* +16 Da, ox, in green), or the disodium adducts of hydrated C_1_-oxidized products (Δ*m/z* +38 Da, ox #, in pink). Oligomers containing GlcAOMe may exchange an additional proton with Na^+^, as is indeed observed (indicated by “+Na”; see signals at 1347 and 1611 in panel e). Yellow shaded signals represent xylooligosaccharides (Xyl), while unshaded labels represent xyloglucan fragments (H_x_P_x_) or cellooligosaccharides (Glc). Note that panels d and e show signals corresponding to the sodium salts of aldonic acids (Δ*m/z* +38 Da, ox #, in pink), indicative of C_1_ oxidation, for both xylan and TXG fragments. Note also that sodium salts of GlcAOMe-containing compounds (Δ*m/z* +38, ox #, in pink) do not necessarily prove C_1_ oxidation, since both the aldonic acid and the GlcAOMe can engage in salt formation. MS data for the other three *Mc*AA9s, analogous to panel e, appear in Fig. S8.

To further explore lytic activity on hemicellulosic substrates, *Mc*AA9s were incubated with tamarind xyloglucan (TXG; *β*-1,4-linked glucose backbone decorated with *α*-1,6-linked xylose moieties on 3 out of 4 glucoses, which can be further appended with β-1,2-linked galactosyl moieties) and birchwood xylan (BX; *β*-1,4-linked xylosyl residues partially replaced with *α*-1,2-linked GlcAOMe). In standard assays, *Mc*AA9A, -B, and -F, but not *Mc*AA9H, were active toward TXG (Fig. 3a). The main products detected on MALDI-TOF had masses corresponding to clusters of oxidized and native xyloglucooligosaccharides with various numbers of hexoses (glucose or galactose) and pentoses (xylose), suggesting that these enzymes can cleave between any two glucosyl residues in the xyloglucan backbone, irrespective of side chains (18, 35, 36) (Fig. 3b and Fig. S7). No signals for the sodium salts of the aldonic acids, which are a hallmark of C_1_ oxidiation, could be detected, indicating that only C_4_ oxidation took place during the degradation of TXG. Even after 24 h of incubation, *Mc*AA9H did not show any activity on TXG (Fig. 3a and c and Fig. S7).

No activity was detected for any of the *Mc*AA9s on BX alone (not shown). Most interestingly, however, coating of cellulose fibers with TXG or BX changed the susceptibility of the hemicelluloses to degradation by the *Mc*AA9s. *Mc*AA9H, which was completely inactive on TXG alone, generated products from TXG in reaction mixtures containing both cellulose and TXG (Fig. 3d), in addition to native cellooligosaccharides (due to background activity) and oxidized xylooligosaccharides generated from PASC. This result suggests that the conformation of the substrate (i.e., free xyloglucan versus xyloglucan interacting with cellulose) is a major determinant of LPMO susceptibility. Furthermore, in contrast to *Mc*AA9A, -B, and -F, signals for the sodium salt of the aldonic acids (Δ*m/z* +38) could be detected, indicating that *Mc*AA9H is able to oxidize the C_1_ position upon cleavage of xyloglucan.

Substrate conformation also seemed to influence activity toward xylan, since all four *Mc*AA9s were, to differing degrees, active toward BX-coated cellulose and produced cellooligosaccharides (from PASC) as well as native and oxidized xylooligosaccharides (from BX or PASC). For *Mc*AA9H, signals corresponding to oxidized xylooligosaccharides (with or without GlcAOMe) were dominating, corroborating the observations for the reactions with PASC alone, which strongly suggested that this enzyme strongly prefers xylan as the substrate (Fig. 3e). On the other hand, for *Mc*AA9A, -B, and -F, xylan coating reduced all lytic activities; cellooligomers were still the dominating products and the signals for xylooligosaccharides were relatively weak, especially for *Mc*AA9A and *Mc*AA9B, which generated only trace amounts of oxidized xylooligomers (Fig. S8a and b).

### Other activities.

Two of the tested LPMOs, *Mc*AA9A and *Mc*AA9F, showed clear activity on cellooligosaccharides (DP6), generating C_4_ oxidation products (Fig. S9a).

One of the enzymes, *Mc*AA9A, displayed trace activity on lichenan, a linear *β*-1,3-*β*-1,4-glucan with a 1:2 ratio of *β*-1,3 to *β*-1,4 linkages (Fig. S9b). None of the *Mc*AA9s was active on ivory nut mannan (INM), a linear *β*-1,4-linked mannan without substitutions, while *Mc*AA9A was able to depolymerize konjac glucomannan (KGM), a linear *β*-1,4-linked mannan with randomly distributed glucosyl residues (∼35%) (37). The MS analysis revealed signals with masses corresponding to native and oxidized hexose oligomers (DP6 to DP12), with or without acetylation (Fig. S9c).

## DISCUSSION

*M. cinnamomea* is a thermophilic, hemicellulose-degrading fungus that grows abundantly on xylan-rich substrates. In our study, we characterized four of the eight AA9s encoded by the fungus (*Mc*AA9A, -B, -F, and -H) and found that the substrate preference of the organism (see Fig. S1 in the supplemental material) (28) is reflected in the substrate preferences of its AA9 LPMOs. All four investigated LPMOs were active on cellulose, as well as different hemicelluloses, to various degrees (Table 1). *Mc*AA9H in the present study is an AA9 LPMO that acts preferentially on xylan, even when xylan is only present in trace amounts, as was the case with PASC as the sole substrate (Fig. 2c).

**TABLE 1:** Summary of *Mc*AA9 activities

Enzyme	Activity on substrate^*a*^:										
	PASC		TXG (TXG fragments ox)	TXG + PASC (TXG fragments ox)	BX (XOSox)	BX + PASC		Cellohexaose (COS ox)	KGM (KGM fragments ox)	Lichenan (lichenan fragments ox)	INM (INM fragments ox)
	COS ox	XOS ox				COS ox	XOS ox				
*Mc*AA9A	+++	−	+++	+++	−	++	+	++	++	+	−
*Mc*AA9B	+++	−	+++	+++	−	++	+	−	−	−	−
*Mc*AA9F	+++	−	+++	+++	−	++	++	++	−	−	−
*Mc*AA9H	+	+++	−	+++	−	−	+++	−	−	−	−

a ox, oxidized; COS, cellooligosaccharides; XOS, xylooligosaccharides; TXG, tamarind xyloglucan; PASC, phosphoric acid swollen cellulose; BX, birchwood xylan; KGM, konjac glucomannan; INM, ivory nut mannan.

So far, LPMO activity on pure xylan has only been reported for two AA9 enzymes. One study described how AA9A from Lentinus similis (*Ls*AA9A) displayed activity toward xylooligosaccharides, although it was at two magnitudes lower than activity on cellooligosaccharides (20). A very recent study showed that an *M. cinnamomea* AA9 (PMO9A_MALCI, or *Mc*AA9G) acted on both cellulose and hemicelluloses equally well, but the available data do not allow an assessment of substrate preferences and background activities in the absence of ascorbic acid were very high in this study (22). One other AA9 that showed weak oxidative activity on xylan when the xylan was combined with cellulose still primarily generated cellooligosaccharides (LPMO9A from *Myceliophthora thermophila* C_1_ [21]). Therefore, the *Mc*AA9H enzyme characterized in the present study stands out due to its clear preference for xylan.

It is intriguing that activity on xylan has been detected for all five *Mc*AA9s studied so far, considering that, previously, only a few LPMOs (two AA9s and two AA14s) have been reported to be able to cleave xylan. Of note, for two of the five *M. cinnamomea* enzymes, *Mc*AA9A and *Mc*AA9B, the xylan activity was weak and could easily have been overlooked. Most studies so far indicate that LPMO activity on xylan is strongly linked to the presence of cellulose, indicating the importance of xylan conformation for susceptibility to LPMOs. Xylan exists in a 3-fold helical screw conformation in solution but flattens into a 2-fold screw ribbon when bound to cellulose microfibrils in the plant cell wall (38–40). This 2-fold screw xylan is closely associated with cellulose and obtains rigidity and packing similar to those of the cellulose microfibrils. The need for a more crystalline substrate can be explained by the general structure of LPMOs, which have relatively flat substrate-binding surfaces that host the histidine brace with the copper ion (41). The fact that most described xylan-active AA9 LPMOs to date require the xylan to be bound to cellulose suggests that they are active on xylan substructures that are found at cellulose-xylan interfaces.

The change from a 3- to 2-fold screw seems to be the precondition for degradation not only by AA9 LPMOs but also by the recently described AA14 LPMOs, which exclusively act on xylan and no other polymers (19). Similar to *Mc*AA9s, AA14 LPMOs were not active on pure xylan but only on birchwood cellulosic fibers consisting of 79% cellulose and 21% xylan. A major difference between *Mc*AA9H and the AA14s is that the AA14s seemed to make very few cuts and that detection of soluble products required addition of xylanases to release short oxidized xylooligomers from the substrate. In contrast, *Mc*AA9H released a wide variety of soluble products on its own, both from PASC and from PASC-xylan mixtures (Fig. 2c and 3). In contrast, the weak activities of *Mc*AA9A and -B on xylan-coated cellulose suggests that these enzymes possess a general ability to target polysaccharides with flat conformations but have only meager affinities for xylan.

While a flat conformation is suggested to be necessary for sufficient interaction with the LPMO active site, several AA9 LPMOs, including *Mc*AA9A, -B, and -F in the present study, act on soluble, noncrystalline substrates, such as cellohexaose and xyloglucan. Similar to xylan, xyloglucan adopts different conformations in solution (twisted conformation) and upon binding to cellulose microfibrils (2-fold helical conformation) (42). Unlike xylan, however, xyloglucan was readily cleaved by *Mc*AA9A, -B, and -F, whereas *Mc*AA9H was only able to attack xyloglucan when it was associated with cellulose (Fig. 3c and d). This conformation dependency for xyloglucan degradation prompts the question of why only *Mc*AA9H required xyloglucan to be in the 2-fold helical conformation for activity. Differences in the substrate-binding surfaces of *Mc*AA9s likely determine the suitability of each LPMO to act on less and more crystalline substrates. Indeed, a simple comparison of structural models of the McAA9s (Fig. S4) shows considerable differences between the surfaces around the active site. The functional implications of these differences would be worth further studies.

With its comparatively few AA9 genes and its ability to grow on a diverse set of substrates, *M. cinnamomea* presents a good model to investigate the specificities of an organism’s full complement of LPMO enzymes. We show here that the differential transcriptional regulation of AA9s previously studied in *M. cinnamomea* during growth on glucose, wheat bran, and beechwood xylan (28) corresponds to the substrate specificities of its LPMOs. *Mc*AA9A was the most consistently expressed under all three conditions, which correlates with its ability to act on the broadest range of substrates tested in the present study (Table 1). *Mc*AA9H, on the other hand, was not at all expressed during growth on glucose but was highly upregulated during growth on xylan, corroborating its role in xylan degradation. Interestingly, for *Mc*AA9C, which was not expressed at all under any condition, we were unable to detect activity in the present study (Fig. S2b). The recently characterized *Mc*AA9G (22) shares 52% sequence identity with *Mc*AA9F and appears in the same clade in the phylogenetic tree (Fig. 1). Therefore, these enzymes are suspected to fulfil similar roles in the organism. Interestingly, however, the gene encoding *Mc*AA9F was highly expressed on both wheat bran and xylan and highly upregulated on both substrates compared to that of glucose, while the one for *Mc*AA9G was very weakly expressed (File S1a) (28).

Our findings contribute to forming a more comprehensive picture of the roles of AA9 LPMOs in an organism, some of which may be targeted toward the decomposition of hemicelluloses or copolymeric plant cell wall structures. Even though activity on cellulose was confirmed for all four investigated *Mc*AA9s, the slow growth of *M. cinnamomea* on cellulosic substrates points to a group of LPMOs that are not used primarily for breaking down cellulose. In their study of xylan-active AA14 LPMOs, Couturier et al. noted that AA14s are primarily present in white- and brown-rot fungi that specifically target the protective heteroxylan-rich layer covering cellulose microfibrils in wood (19). LPMOs such as *Mc*AA9H in *M. cinnamomea*, and possibly AA9 LPMOs in other ascomycetes, might therefore fill a role similar to the role of AA14s in basidiomycetes.

In conclusion, our study sheds light on the role of AA9s in hemicellulose degradation by *M. cinnamomea* in nature and has revealed at least one LPMO, *Mc*AA9H, with unprecedented properties. More fundamental biochemical and genetic studies are still needed to fully unravel the joint catalytic potential and biological function of complete sets of fungal LPMOs in different species. It is important to note that aside from providing the organism with a multitude of catalytic capabilities and targeting multiple locations in the plant cell wall, LPMO multiplicity could also relate to other factors that have not been addressed here. In particular, LPMOs may vary in terms of their interactions with the nonenzymatic or enzymatic reductants that they need for activity (43, 44), and the presence of such reductants will vary between ecological niches and fungal growth stages.

## MATERIALS AND METHODS

### Strains and growth conditions.

*Malbranchea cinnamomea* strain FCH 10.5 (CBS 143691) (45) was used as a source of LPMO genes, and Escherichia coli strain Stellar (TaKaRa) and Pichia pastoris strain SMD1168H (Invitrogen) were used as hosts for subcloning and heterologous expression of recombinant proteins, respectively.

*M. cinnamomea* was grown in liquid basal medium containing 4 g liter^−1^ KH_2_PO_4_, 13.6 g liter^−1^ (NH_4_)_2_SO_4_, 0.8 g liter^−1^ CaCl_2_·2H_2_O, 0.6 g liter^−1^ MgSO_4_·7H_2_O, 6 g liter^−1^ Bacto peptone, 10 mg liter^−1^ FeSO_4_·7H_2_O, 3.2 mg liter^−1^ MnSO_4_·H_2_O, 2.8 mg liter^−1^ ZnSO_4_·7H_2_O, 4 mg liter^−1^ CoCl_2_·6H_2_O, and 200 ml liter^−1^ Tween 80. The pH was adjusted to 5.8, and 1% (wt/vol) of a carbon source (wheat bran, Avicel, filter paper, or carboxymethyl cellulose [CMC]) was added before autoclaving. Volumes of 125 ml liquid medium in baffled Erlenmeyer flasks (500 ml) were inoculated with a spore suspension prepared from fresh malt extract agar (MAE) plates. Cultures were grown at 45°C for 19 days with shaking at 250 rpm, and samples were taken at regular intervals.

### Sequence analysis and homology modeling.

PRALINE (http://www.ibi.vu.nl/programs/pralinewww/), Clustal Omega (https://www.ebi.ac.uk/Tools/msa/clustalo/), and MEGA7 software were used for multiple-sequence alignment (MSA) of AA9 proteins. Phylogeny.fr (http://www.phylogeny.fr/) and iTOL (https://itol.embl.de/) were used for generating phylogenetic trees. A detailed list of proteins used in the alignments is provided in File S1d. For homology modeling, the online tool Phyre2 was used with default parameters (31). A list of templates used for modeling is provided in File S1e.

### Cloning of genes encoding *Mc*AA9s.

Eight putative AA9 genes are encoded by the *M. cinnamomea* genome (strain FCH 10.5; publicly available at DDBJ/EMBL/GenBank under the assembly accession no. FQSS00000000) and were named according to current naming conventions: *mclpmo9A* (locus tag MALCI_0000123.8), *mclpmo9B* (MALCI_0000604.52), *mclpmo9C* (MALCI_0000604.99), *mclpmo9d* (MALCI_630.43), *mclpmo9e* (MALCI_0000639.3), *mclpmo9f* (MALCI_0000739.16), *mclpmo9g* (MALCI_0000780.38), and *mclpmo9h* (MALCI_0000638.10) (File S1f and g). Total RNA was extracted from *M. cinnamomea* FCH 10.5 grown in fungal basal medium containing 1% wheat bran as the sole carbon source for 48 h, as described previously (28). Three micrograms of RNA was used for cDNA synthesis in a 20-μl reaction mixture containing 1 μl Revert Aid H minus reverse transcriptase (R0191; Thermo Fisher), 0.5 μl RiboLock (EO0381; Thermo Fisher), and 2 μl 10 mM deoxynucleoside triphosphate (dNTP) mix (EP0451; Thermo Fisher).

Target genes were amplified from cDNA in 20-μl PCRs using the Phusion high-fidelity PCR kit (F553L; Thermo Fisher) with the following components: 4 μl 5× HF buffer, 0.4 μl 10 mM dNTPs, 0.5 μM each primer, 0.8 μl cDNA synthesis product, 0.2 μl Phusion polymerase. PCR cycle conditions were 98°C for 2 min; 30 cycles of 98°C for 10 s, 60°C for 30 s (−0.5°C every cycle), and 72°C for 1 min; and 72°C for 10 min for the final extension. Amplification of the expected products was verified on agarose gel electrophoresis, and DNA fragments were extracted by a GeneJET gel extraction kit (K0692; Thermo Fisher). Primers were designed to contain 3′ and 5′ flanking regions homologous to the desired insertion points in the pPICZαA vector, as well as BstBI and SalI restriction enzyme recognition sites. Since the N-terminal histidine is essential for LPMO functionality, *M. cinnamomea lpmo9* full-length genes were cloned with their intact secretion signal into the Pichia pastoris pPICZαA expression vector. To avoid possible interference of a protein tag, the native stop codons were included in the gene sequence to prevent the addition of the C-terminal His_6_ tag from pPICZαA. A list of the primers used is provided in File S1h. Vector pPICZαA was linearized by digestion with the restriction enzymes EcoRI-FD and SalI-FD (Thermo Fisher) before being used in the In-Fusion cloning reaction (638909; TaKaRa) with PCR amplicons according to the manufacturer’s protocol. Supplied competent E. coli Stellar cells were transformed by heat shock with 2 μl of In-Fusion mix and positive transformants selected on medium containing zeocin. The presence of recombinant genes was checked by colony PCR, and completeness was verified by sequencing (Eurofins).

### Heterologous expression and purification of *Mc*AA9s.

Transformation of competent P. pastoris SMD1168H (Invitrogen) was performed, as described in the manufacturer’s manual, with MssI-linearized pPICZαA recombinant plasmids. Zeocin-resistant transformants were used for small-scale protein expression in 96-well deep-well microplates. The presence of recombinant protein was confirmed by SDS-PAGE, and the clones with the highest expression levels were used for large-scale protein production in a fed-batch process in bioreactors (Infors) according to the *Pichia* fermentation process guidelines (Invitrogen). Briefly, 500 ml fermentation basal salts medium containing PTM_1_ trace salts, 4% glycerol, and 0.003% antifoam was inoculated with 50 ml P. pastoris preculture. The pH was kept at 5.6 with 28% ammonium hydroxide throughout the whole fermentation. After about 24 h of batch growth, glycerol feed was initiated for 24 h until wet cell weight was above 180 mg ml^−1^. Gene expression under the control of the AOX1 promoter was induced by switching to a 100% methanol feed, first at a low feed rate (1 to 3 ml h^−1^) for several hours to allow for cell adaptation and then a higher rate (2 to 6 ml h^−1^). Dissolved oxygen values were kept above 20% by controlling the feed rate, agitation, and aeration. After 4 to 5 days of induction, culture broth containing the secreted recombinant protein was separated from the cells by centrifugation and filtering.

Protein purification was done by anion-exchange chromatography (AEX) alone (*Mc*AA9A, -B, and -F) or in combination with hydrophobic interaction chromatography (HIC) (*Mc*AA9C and -H) on an ÄKTA system (GE Healthcare). For AEX, culture broth was concentrated and buffer exchanged to 20 mM Tris-HCl, pH 8.0, using a cross-flow filtration capsule (Pall Minimate TFF) with a 10-kDa-cutoff polyethersulfone (PES) membrane. To purify enzymes using AEX, a HiTrap QLX 1-ml column (GE Healthcare) was used with start buffer (A; 20 mM Tris-HCl, pH 8.0) and elution buffer (B; 20 mM Tris-HCl, pH 8.0, 1 M NaCl) at a flow rate of 1 ml min^−1^ and a gradient of 0 to 100% B over 60 min. Fractions containing target protein were subjected to a further step of HIC with a HiLoad 16/10 phenyl Sepharose column (GE Healthcare) using start buffer [A; 50 mM Tris-HCl, pH 8.0, 2 M (NH_4_)_2_SO_4_] and elution buffer (B; 50 mM Tris-HCl, pH 8.0) with a flow rate of 2 ml min^−1^ and a stepwise gradient of 40%, 80%, and 100% B. Proteins with electrophoretic purity were concentrated using 3-kDa-cutoff spin columns (Pierce protein concentrator PES; Thermo Fisher), buffer exchanged to 20 mM sodium citrate buffer, pH 6.0, and stored in aliquots at –20°C until further use. The concentration of purified proteins was determined spectrophotometrically by NanoDrop using calculated molecular mass and molar extinction coefficients derived from the sequence. The purified enzymes were treated with EndoHf (New England Biolabs) under denaturing conditions, according to the manufacturer’s instructions, and analyzed using SDS-PAGE to determine N-glycosylation.

### Enzyme reactions.

The following substrates were used: phosphoric acid swollen cellulose (PASC; prepared from Avicel PH-101 as described in reference 46), cellohexaose, tamarind xyloglucan (TXG), ivory nut mannan (INM), barley β-glucan, konjac glucomannan (KGM), lichenan (all from Megazyme), and birchwood and beechwood xylan (Sigma-Aldrich). The substrates (0.2 to 0.5%, wt/vol) were incubated with purified enzyme (1 to 3 μM) and 1 mM ascorbic acid in 50 mM Bis-Tris buffer, pH 6.1, in a total volume of 200 μl, at 40°C with shaking at 1,000 rpm, for 4 h. LPMO activity was stopped by adding 1 volume of 0.2 M HCl and keeping the samples at 4°C until analysis. Control reactions were set up without ascorbic acid. As positive controls, Neurospora crassa LPMO9C (*Nc*LPMO9C) (23) and Thermoascus aurantiacus LPMO9A (*Ta*LPMO9A) (47), both produced in P. pastoris, were used. For time course experiments, 50-μl aliquots were removed from reaction mixtures (total initial volume, 400 μl) at defined time points, and the reaction was stopped by adding 50 μl 0.2 M HCl. Before analysis, all samples were filtered with 0.45-μm-pore-size 96-well filter plates (Millipore) and a vacuum manifold.

### Detection of LPMO activity. (i) Measurement of H_2_O_2_.

Generation of hydrogen peroxide by LPMOs was detected using the Amplex Red assay (29), which employs Ampliflu Red (10-acetyl-3,7-dihydroxyphenoxazine; 90101; Sigma-Aldrich) and horseradish peroxidase (HRP; P8125; Sigma-Aldrich). A 200-μl reaction mixture contained 0.05 mM Ampliflu Red, 3.55 U ml^−1^ HRP, 0.05 mM ascorbic acid (AscA), and 0.4 to 3.3 μM enzyme in 50 mM sodium citrate buffer, pH 6.0. The reaction was measured at 30°C for 30 min on a FLUOstar Omega microplate reader by recording fluorescence (excitation, 544 nm; emission, 600 nm) every 60 s. A standard curve was prepared with H_2_O_2_ (0.25 to 5 μM).

### (ii) Analysis of oxidized and nonoxidized oligosaccharides

Enzyme reaction products were analyzed by high-performance anion-exchange chromatography with pulsed amperometric detection (HPAEC-PAD) and matrix-assisted laser desorption/ionization time-of-flight mass spectrometry (MALDI-TOF MS). Analysis with HPAEC-PAD was performed on a Dionex ICS5000 instrument equipped with a CarboPac PA1 guard column (2 by 50 mm; Dionex) and a CarboPac PA1 column (2 by 250 mm; Dionex). Two mobile phases were used, A (0.1 M NaOH) and B (1 M sodium acetate in 0.1 M NaOH). For analysis of cellulosic substrates, a 50-min gradient (48) was used. The flow rate was set to 0.25 ml min^−1^ with the following gradients: 0 to 10 min, 0 to 10% B (curve 5); 10 to 35 min, 10 to 30% B (curve 5); 35 to 40 min, 30 to 100% B (curve 6); 40 to 41 min, 100 to 0% B (curve 6); 41 to 50 min, 100% A (curve 5). For analysis of hemicellulosic substrates, a 75-min gradient (18) was used: 0 to 35 min, 0 to 10% B (curve 5); 35 to 60 min, 10 to 30% B (curve 5); 60 to 65 min, 30 to 100% B (curve 6); 65 to 66 min, 100 to 0% B (curve 6); 66 to 75 min, 100% A (curve 5). Soluble cellooligosaccharides (DP1-6; Megazyme) and xylooligosaccharides (DP1-4; Megazyme) were used as standards. Chromatograms were recorded and analyzed with Chromeleon software.

Analysis by MALDI-TOF was performed on an Ultraflex instrument (Bruker Daltonics) equipped with a nitrogen 337-nm laser beam in positive reflector mode, as described previously (18). A 1.5-μl sample was mixed with 1.5 μl matrix solution (10 mg ml^−1^ 2,5-dihydroxybenzoic acid in 30% acetonitrile and 0.1% trifluoroacetic acid), applied to an MTP 384 ground steel target plate (Bruker Daltonics), and air dried. Data were collected with flexControl software (Bruker) and analyzed using mMass software.

### Data availability.

The nucleotide sequences of the genes (genomic DNA) encoding (putative) *Mc*AA9s have been deposited in the GenBank database under the following accession numbers: *Mc*AA9A, *mclpmo9a*, MK135883; *Mc*AA9B, *mclpmo9b*, MK135884; *Mc*AA9C, *mclpmo9c*, MK095629; *Mc*AA9D, *mclpmo9d*, MK135885; *Mc*AA9E, *mclpmo9e*, MK135886; *Mc*AA9F, *mclpmo9f*, MK135887; *Mc*AA9G, *mclpmo9g*, MK135888; and *Mc*AA9H, *mclpmo9h*, MK135889.

## Supplementary Material

Supplemental file 1

Supplemental file 2
