# Supplementary material for: Specific Xylan Activity Revealed for AA9 Lytic Polysaccharide Monooxygenases of the Thermophilic Fungus Malbranchea cinnamomea by Functional Characterization
Source: Appl Environ Microbiol. 2019 Nov 14;85(23):e01408-19. doi: 10.1128/AEM.01408-19 (PMC6856335; doi:10.1128/AEM.01408-19)
Supplement: Supplemental file 1 [file AEM.01408-19-s0001.pdf]

Supplemental Figures S1-S9

Functional characterization of AA9 LPMOs in the thermophilic fungus *Malbranchea cinnamomea* reveals specific xylan activity

Silvia Hüttner[a,b], Anikó Várnai[c], Dejan M. Petrović[c], Cao Xuan Bach[d], Dang Thi Kim Anh[d], Vu Nguyen Thanh[d], Vincent G. H. Eijsink[c], Johan Larsbrink[a,b], Lisbeth Olsson[a,b,#]

Affiliations
[a] Department of Biology and Biological Engineering, Division of Industrial Biotechnology, Chalmers University of Technology, Gothenburg, Sweden
[b] Wallenberg Wood Science Center, Chalmers University of Technology, Gothenburg, Sweden
[c] Faculty of Chemistry, Biotechnology and Food Science, Norwegian University of Life Sciences (NMBU), Ås, Norway
[d] Centre for Industrial Microbiology, Food Industries Research Institute, Thanh Xuan, Hanoi, Vietnam

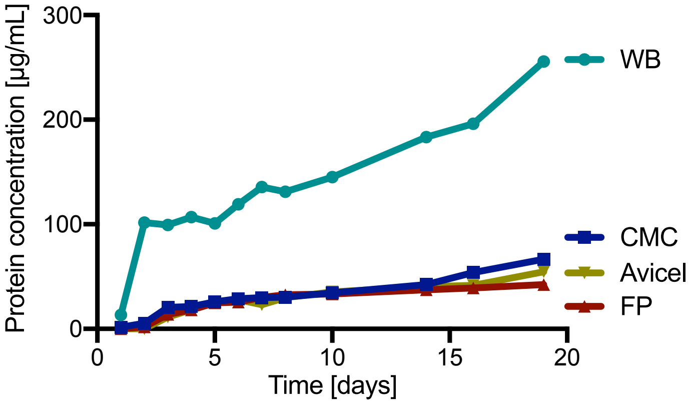

Supplemental Figure S1. Growth of *M. cinnamomea* on different carbon sources. *M. cinnamomea* FCH 10.5 was grown from spores in liquid medium containing 1% of a single carbon source over 19 days. Extracellular protein concentration in the medium (measured by Bradford assay) was used as a proxy to determine growth of mycelium. WB, wheat bran; CMC, carboxymethyl cellulose; FP, filter paper.

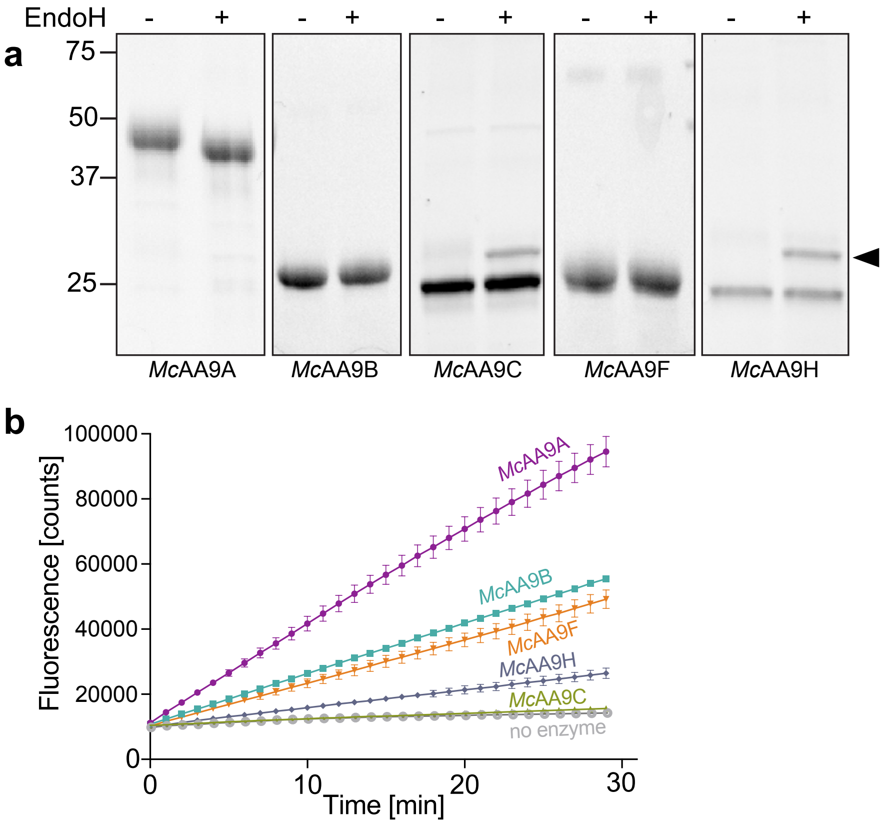

Supplemental Figure S2. Purified *Mc*AA9s separated by SDS-PAGE and generation of hydrogen peroxide. a The five *Mc*AA9s *Mc*AA9A, B, C, F and H were purified to homogeneity and analyzed by SDS-PAGE. Glycosylation was assessed by studying the effect of EndoH digestion on electrophoretic mobility, and for each LPMO the gels show an untreated (-) and a treated (+) sample. The results indicate the presence of N-glycans on *Mc*AA9A only. No indication for N-glycosylation was detected for *Mc*AA9C despite one predicted N-glycosylation site (Asn184). The band indicated by the black arrowhead is the EndoH enzyme which was visible upon longer exposure of the gel. **b** The progression of H_2O_2 production by purified enzymes during the Amplex Red assay was followed for 30 min at 30°C by monitoring fluorescence (excitation: 544 nm, emission: 600 nm). Samples contained 0.83 µM enzyme (for *Mc*AA9A, B, C, F) or 0.415 µM enzyme (for *Mc*AA9H), 0.05 mM Ampliflu Red, 3.55 U mL HRP and 0.05 mM ascorbic acid in 50 mM sodium citrate buffer, pH 6.0. Reactions were performed in triplicates; error bars represent standard deviations.

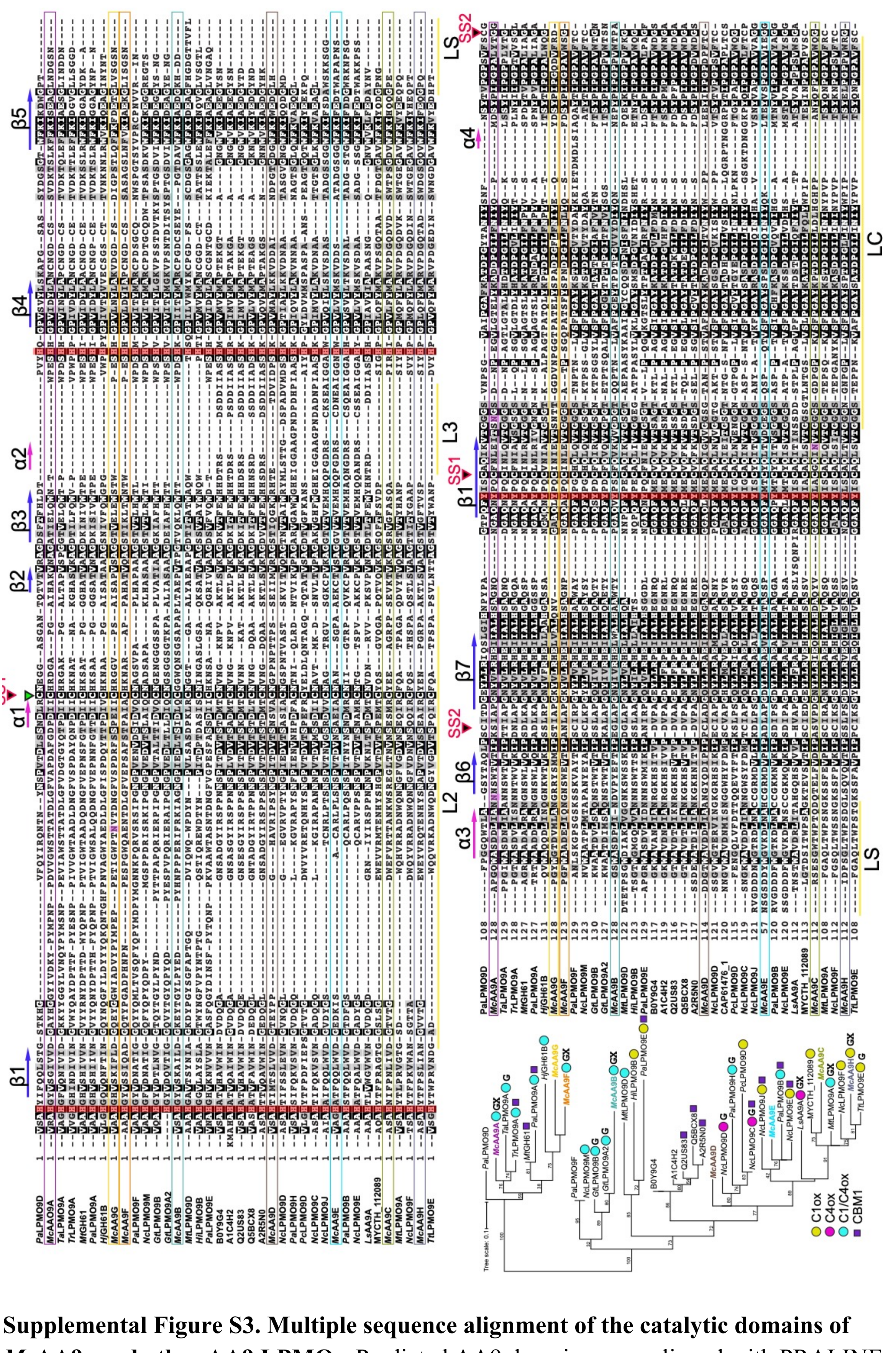

Supplemental Figure S3. Multiple sequence alignment of the catalytic domains of *Mc*AA9s and other AA9 LPMOs. Predicted AA9 domains were aligned with PRALINE multiple sequence alignment (http://www.ibi.vu.nl/programs/pralinewww/) using a PSI-

BLAST strategy. Residues or regions were annotated based on the alignment and/or based on publications. Residues that are conserved in more than 50% of the sequences are shown in white on black; grey shading indicates that more than 50% of residues in the corresponding column exhibit similar physico-chemical properties. The red shading indicates conserved residues that directly coordinate the copper (H, H) and help in shaping the copper site (Y). The secondary structure assignments (α-helices indicated as magenta arrows, β-strands indicated as blue arrows, loop regions L2, L3, LS and LC regions that contribute to shaping the substrate-binding surface in yellow) refer to *Nc*LPMO9C and were determined in (Borisova et al., 2015, J Biol Chem 290:22955–22969). Red-black arrowheads (labelled SS1 and SS2) indicate cysteine residues involved in disulfide bond formation. The asparagines of predicted N-glycosylation sites (in *Mc*AA9A, C and G) are shaded in pink. The phylogenetic tree is the same as in Fig. 1, and is there to visualize the phylogenetic relationship of the aligned sequences to each other. For details see Supplemental File 1d.

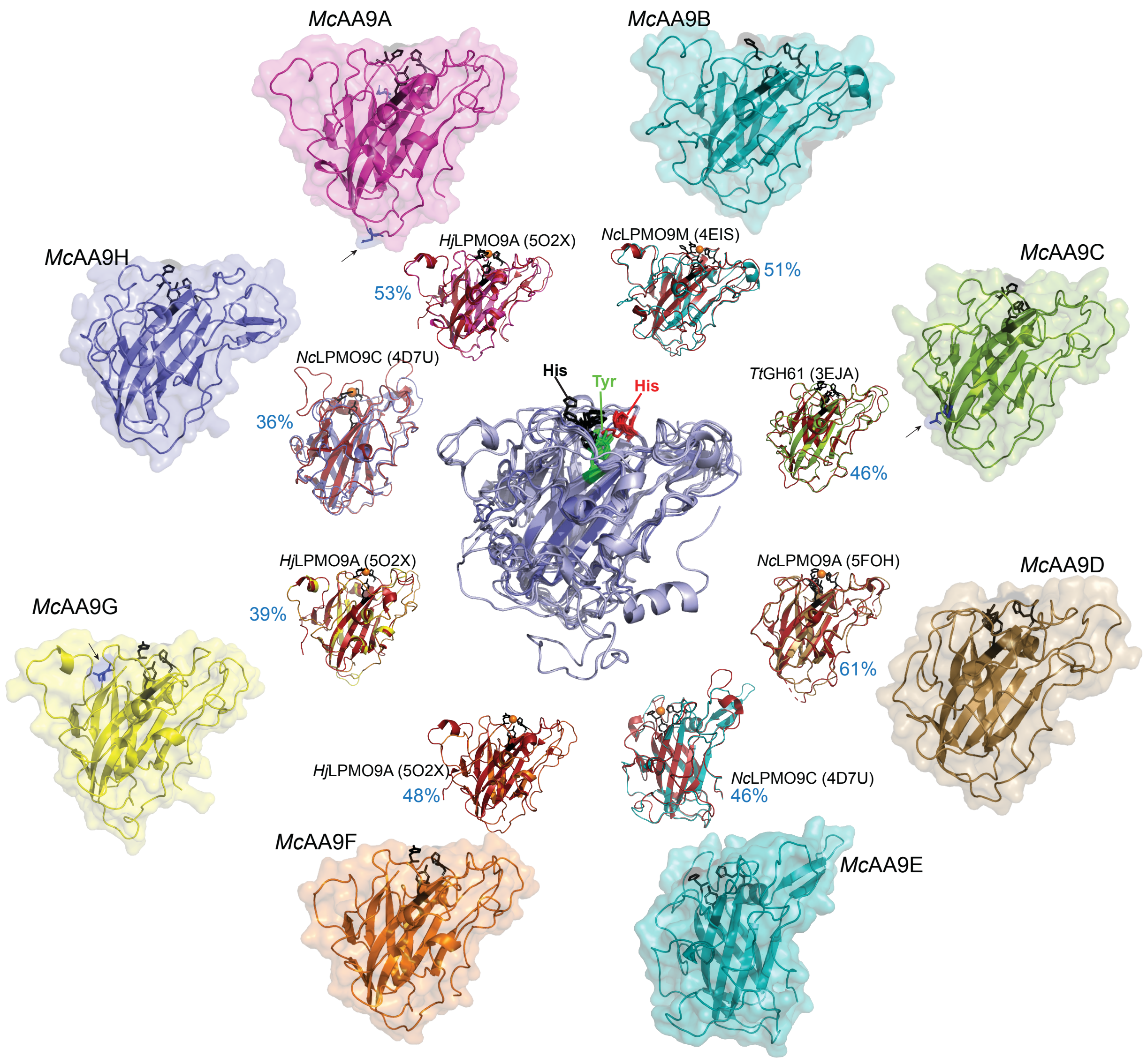

Supplemental Figure S4. Models of *Mc*AA9s aligned with their top modelling template. Homology modelling of catalytic domains of *Mc*AA9s was performed with the Phyre2 web portal (http://www.sbg.bio.ic.ac.uk/phyre2). The outer ring in the figure shows the predicted structures with the amino acid side chain of the histidine brace (H-H-Y) shown as black sticks. The side chains of residues that are putatively N-glycosylated are shown as blue sticks and marked by black arrows. The middle ring of the figure shows the *Mc*AA9 models aligned with their top modelling template, i.e. the closest related AA9 LPMO with an experimentally determined structure. The name of the LPMO and the corresponding PDB code are written next to the aligned protein structures, as well as, in blue, the percentage identity of a pairwise sequence alignment between the template and the respective *Mc*AA9. The copper ion is shown as an orange sphere. The structures in the center show an alignment of all *Mc*AA9 homology models. The amino acid residues of the histidine brace (H-H-Y) all align very well in the homology models. For details concerning the Phyre2 results, see Supplemental File 1e.

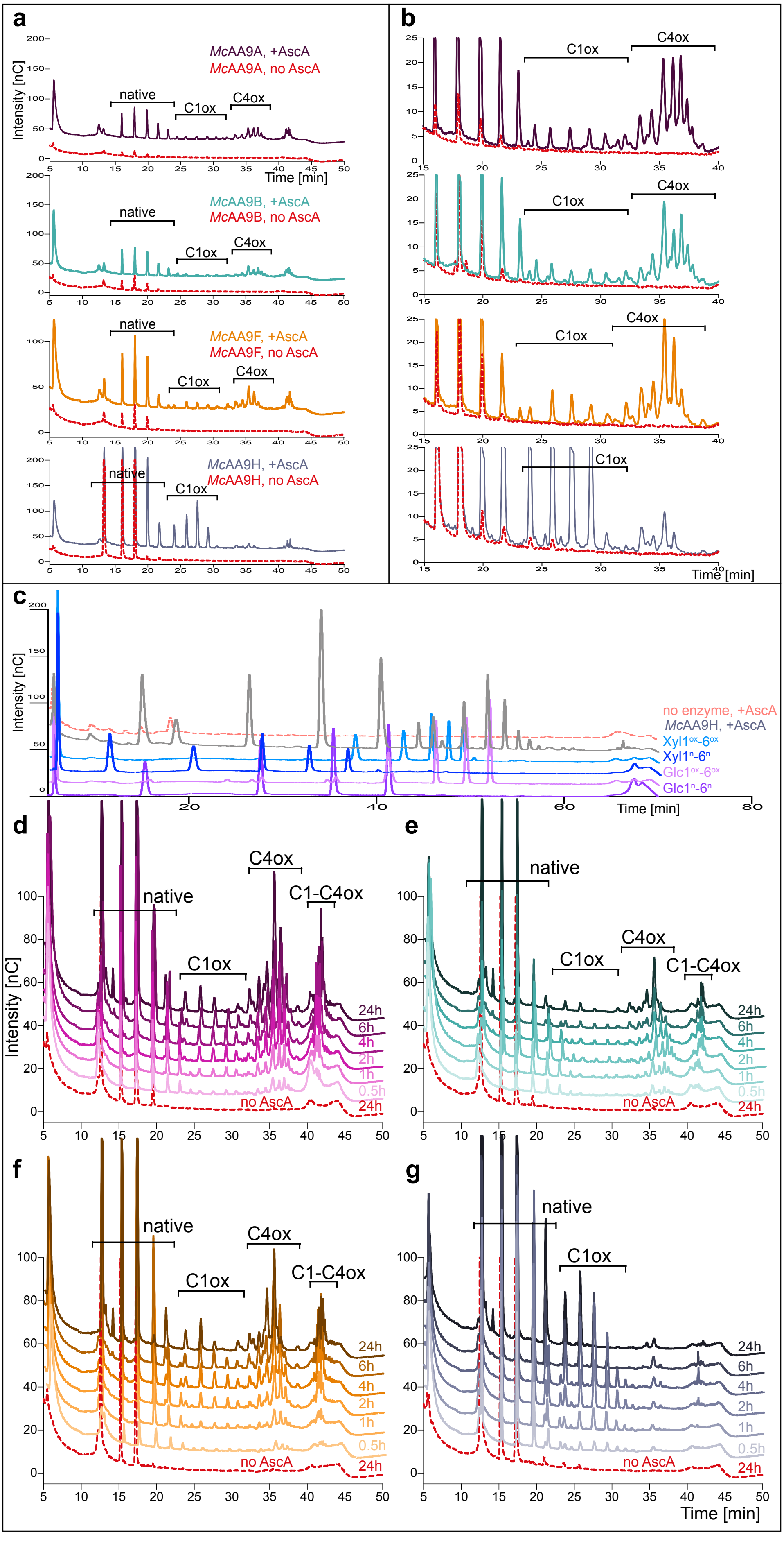
a
b
c
d
e
f
g
Intensity [nC]
Time [min]
McAA9A, +AscA
McAA9A, no AscA
McAA9B, +AscA
McAA9B, no AscA
McAA9F, +AscA
McAA9F, no AscA
McAA9H, +AscA
McAA9H, no AscA
native
C1ox
C4ox
C1-C4ox
no enzyme, +AscA
McAA9H, +AscA
Xyl1ox-6ox
Xyl1n-6n
Glc1ox-6ox
Glc1n-6n
24h
6h
4h
2h
1h
0.5h
no AscA

Supplemental Figure S5. Activity of *Mc*AA9s on PASC. HPAEC-PAD elution patterns of soluble products generated from phosphoric acid swollen cellulose (PASC) after incubation with *Mc*AA9s. Panel **a** shows peaks eluting at 5-50 min, whereas panel **b** shows a zoom-in to 15-40 min, when peaks representing C1-oxidized and C4-oxidized cello-oligosaccharides elute. Panel **c** compares the elution pattern of PASC + *Mc*AA9H with a no-enzyme control, as well as with standards of native and oxidized cello- and xylo-oligosaccharides. **d-f** show HPAEC-PAD elution chromatograms after incubation with *Mc*AA9s for varying amounts of time. Enzymes (1 μM) were incubated in 50 mM BisTris buffer, pH 6.1, with 0.3% (w/v) PASC at 40°C with (+AscA) or without (no AscA) 1 mM ascorbic acid. Samples were taken after 4h (a, b) or at indicated time points between 0 and 24h (c-f). **c** *Mc*AA9A, **d** *Mc*AA9B, **e** *Mc*AA9F, **f** *Mc*AA9H. Native and oxidized products are indicated in the figure and these product assignments were based on previous work (Isaksen et al., 2014, J Biol Chem 289:2632–2642.). Note that some native products appear in samples from reactions without AscA, indicative of minor hydrolytic background activities in the LPMO samples. For panel **c** only, the 75-min HPAEC-PAD program was used.

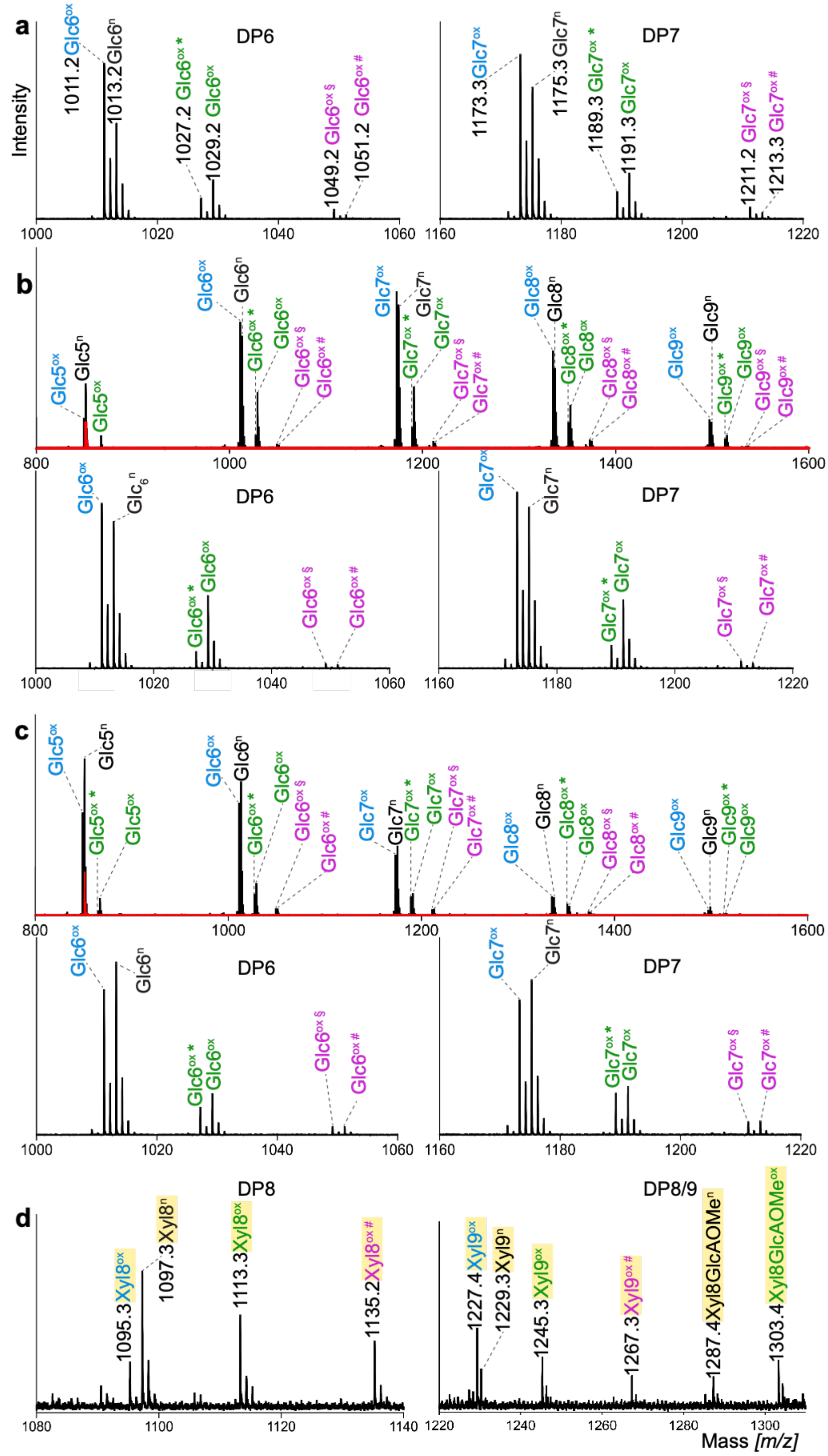

Supplemental Figure S6. Activity of *Mc*AA9s on cellulose. MALDI-TOF MS spectra showing the sodium adducts of reaction products generated from phosphoric acid swollen cellulose (PASC) after incubation with *Mc*AA9s. Enzymes (1 μM) were incubated in 50 mM

BisTris buffer, pH 6.1, and 0.3% (w/v) PASC for 4h at 40°C with or without 1 mM ascorbic acid. **a** *Mc*AA9A zoom-in to DP6 and DP7 (for full spectrum, see Fig. 2b). **b** *Mc*AA9B full spectrum, with (black) and without (red) ascorbic acid, and zoom-in to DP6 and DP7. **c** *Mc*AA9F full spectrum, with (black) and without (red) ascorbic acid, and zoom-in to DP6 and DP7. **d** *Mc*AA9H zoom-in to DP8 and DP8/9 (for full spectrum, see Fig. 2c). Labelled peaks show the masses of monosodium adducts of native oligosaccharides (n, in black), C1-oxidized lactone or C4-oxidized ketoaldose species (Δ*m/z* -2 Da, ox, in blue), hydrated C1-oxidized aldonic acid or C4-oxidized gemdiol species (Δ*m/z* +16 Da, ox, in green), hydrated double oxidized species (Δ*m/z* +14, ox *, in green), the disodium adducts of hydrated double oxidized oligosaccharides (Δ*m/z* +36 Da, ox §, in pink) or the disodium adducts of hydrated C1-oxidized products (Δ*m/z* +38 Da, ox #, in pink). Labels for signals corresponding to xylo-oligosaccharides are shaded in yellow. Note that sodium salts of GlcAOMe-containing compounds (ox #, in pink) do not necessarily proof C1 oxidation, since both the aldonic acid and the GlcAOMe can engage in salt formation. DP, degree of polymerisation.

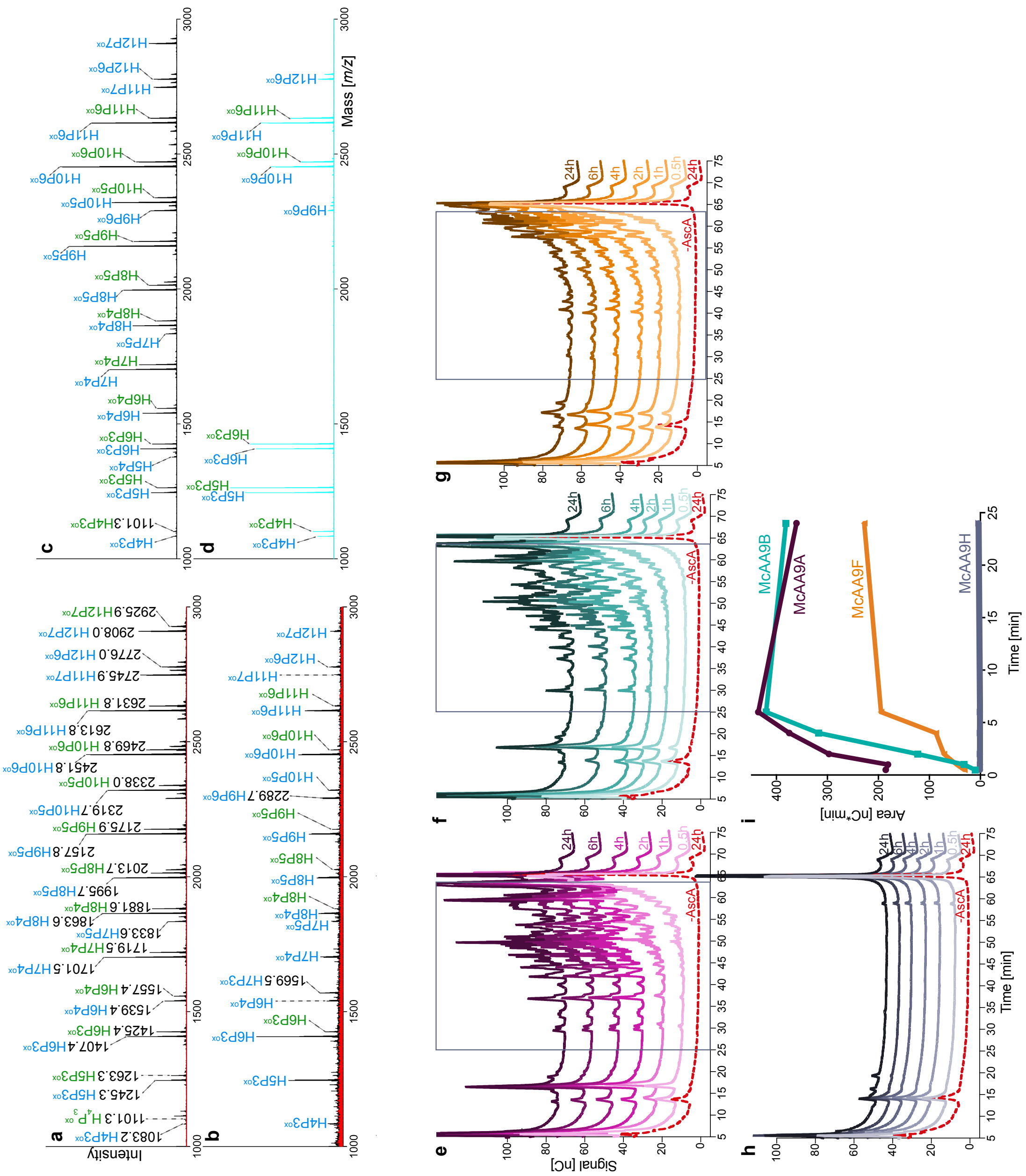

Supplemental Figure S7. Activity of *Mc*AA9s and control LPMOs on TXG. a-d MALDI-TOF MS spectra highlighting the sodium adducts of reaction products generated from tamarind xyloglucan (TXG) after incubation with *Mc*AA9B (**a**), *Mc*AA9F (**b**), and the control LPMOs *Ta*LPMO9A (**c**, black) and *Nc*LPMO9C (**d**, cyan). Enzymes (1 µM) were incubated in 50 mM BisTris buffer, pH 6.1, containing 0.3% (w/v) TXG for 3.5h at 40°C with 1 mM ascorbic acid (black or cyan spectra) or without ascorbic acid (red spectra). Labelled peaks represent C1-oxidized lactone or C4-oxidized ketoaldose species (Δ*m/z* -2 Da, ox, in blue) and the corresponding aldonic acid or gemdiol species (Δ*m/z* +16 Da, ox, in green). *Nc*LPMO9C (**d**) shows a pattern that is characteristic for cleavage at the nonreducing end of non-substituted glucosyl residues only. This results in a peak profile with clusters of xyloglucan fragments where the number of xylose residues is a multiple of three, since in TXG a non-substituted glucosyl unit generally appears every fourth residue in the xyloglucan backbone (Agger et al., 2014, Proc Natl Acad Sci 111:6287–6292.). The product profiles for *Mc*AA9B (**a**), *Mc*AA9F

(**b**) and *Ta*LPMO9A (**c**) show that cleavage occurs both between substituted and unsubstituted glucosyl residues. MS data for *Mc*AA9A and *Mc*AA9H is shown in Fig. 3. H, hexose; P, pentose. **e-i** HPAEC-PAD elution patterns of product formation over time in reactions with TXG after incubation with *Mc*AA9A (**e**), *Mc*AA9B (**f**), *Mc*AA9F (**g**) or *Mc*AA9H (**h**). By quantifying the areas of peaks eluting between 25 and 63 minutes (= TXG-degradation-specific peaks), product formation of *Mc*AA9s on TXG over time was estimated (**i**). Reaction mixes contained enzyme (3 μM for Mc9A, B, F, 1.35 μM for Mc9H) in 50 mM BisTris buffer, pH 6.1, with 0.3% (w/v) TXG with or without 1 mM ascorbic acid (AscA) and were incubated at 40°C. In the absence of AscA (dashed red line, –AscA), no peaks could be detected between 15 and 65 minutes, whereas reactions with AscA (solid lines) yielded a multitude of peaks corresponding to native and oxidized xyloglucan-fragments (Agger et al., 2014), except for *Mc*AA9H.

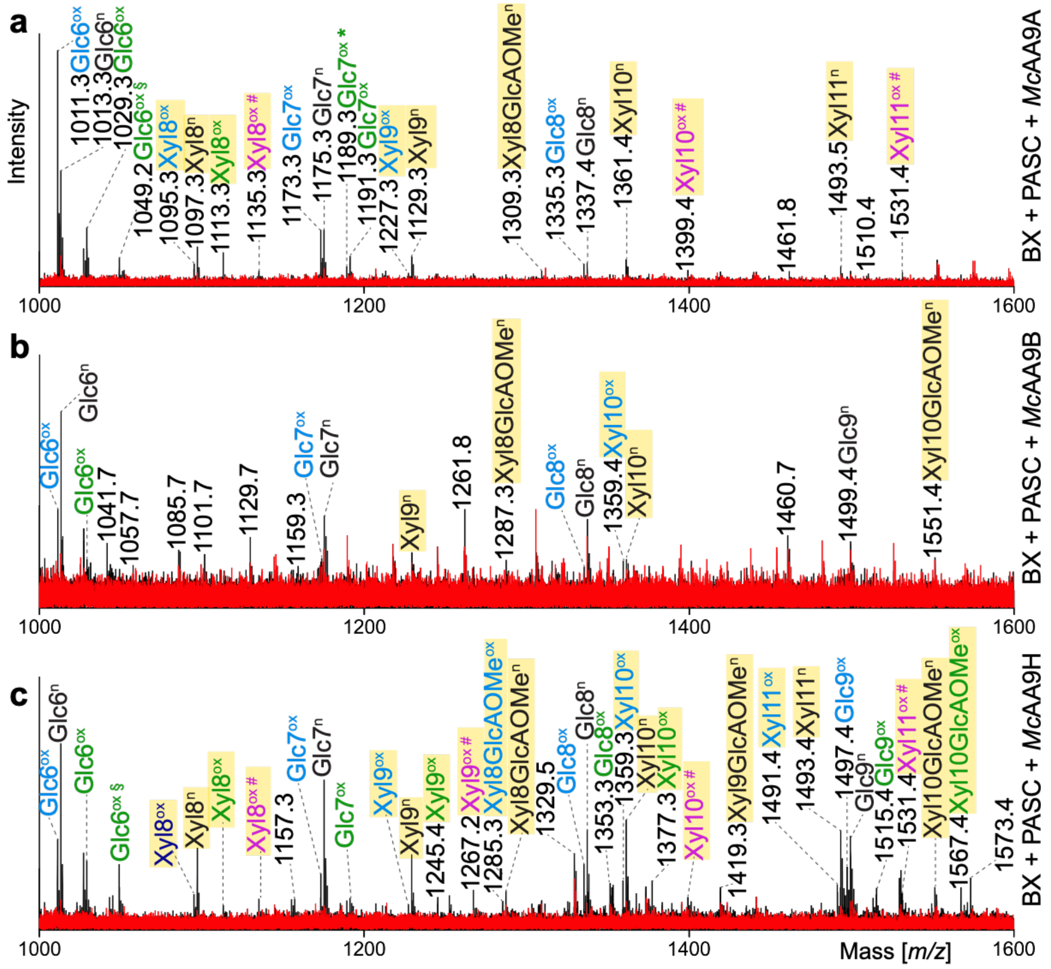

Supplemental Figure S8. Activity of *Mc*AA9s on xylan-coated cellulose. MALDI-TOF MS spectra highlighting the sodium adducts of reaction products generated upon incubation of a mixture of birchwood xylan (BX) and PASC with *Mc*AA9A (**a**), *Mc*AA9B (**b**) or *Mc*AA9F (**c**), in reactions with 1 mM ascorbic acid (black spectra) or without ascorbic acid (red spectra). Reactions contained 3 μM enzyme and 0.15% (w/v) each of birchwood xylan and PASC in 50 mM BisTris buffer, pH 6.1, and were incubated for 4h at 40°C. Labelled peaks show the masses of monosodium adducts of native oligosaccharides (n, in black), C1-oxidized lactone or C4-oxidized ketoaldose species (Δ*m/z* -2 Da, ox, in blue), hydrated C1-oxidized aldonic acid or C4-oxidized gemdiol species (Δ*m/z* +16 Da, ox, in green), hydrated double oxidized species (Δ*m/z* +14, ox *, in green) and the disodium adducts of hydrated C1-oxidized products (Δ*m/z* +38 Da, ox #, in pink). MS data for *Mc*AA9H is shown in Fig. 3e.

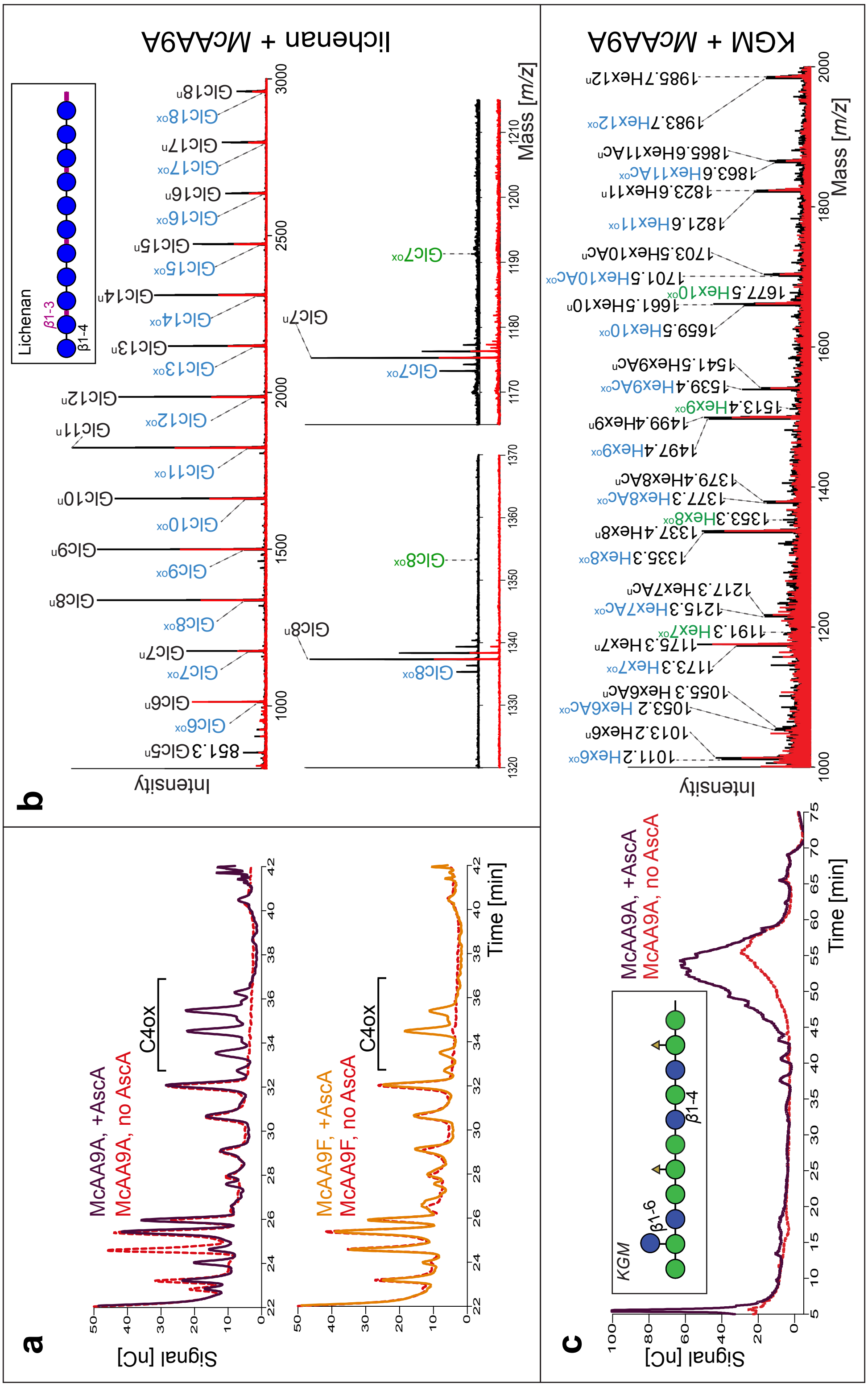

Supplemental Figure S9. Activity of *Mc*AA9s on cellohexaose, konjac glucomannan (KGM) and lichenan. a HPAEC-PAD elution patterns of soluble products generated from cellohexaose after incubation with *Mc*AA9A or *Mc*AA9F between 22 and 42 min, when peaks representing C1-oxidized and C4-oxidized cello-oligosaccharides elute. Enzymes (3 µM) were incubated in 50 mM BisTris buffer, pH 6.1, with 0.3% (w/v) cellohexaose for 3.5h at 40°C with (+AscA) or without (no AscA) 1 mM ascorbic acid. Only in the presence of ascorbic acid, cello-oligosaccharides oxidized at the C4 position (C4ox) could be detected.

Note that the peaks present in both +AscA and no AscA traces likely stem from contaminations in the cellohexaose sample, as well as minor hydrolytic background activities in the LPMO samples. **b** MALDI-TOF MS spectra of lichenan after incubation with *Mc*AA9A, with ascorbic acid (+AscA, black spectra) or without ascorbic acid (no AscA, red spectra). The insert in panel **b** shows the putative structure of lichenan: glucose (blue circles) β-1,3- or β-1,4-linked. Enzymes (3 μM) were incubated in 50 mM BisTris buffer, pH 6.1, with 0.3% (w/v) lichenan for 4h at 40°C with or without 1 mM ascorbic acid. In the presence of ascorbic acid, oxidized cello-oligosaccharides could be detected. Note that the signals for native oligosaccharides represent shorter lichenan or isolichenan fragments that are present in the sample even without enzyme action. Signals for oxidized oligosaccharides represent oxidation products from the cleavage of longer lichenan fragments by LPMOs. Signals on MALDI-TOF MS spectra corresponding to masses of native and oxidized cello-oligosaccharides (Glc) are labelled as follows: native (n, in black), C1-oxidized lactone or C4-oxidized ketoaldose species (Δ*m/z* -2 Da, ox, in blue), or the corresponding aldonic acid or gemdiol forms (Δ*m/z* +16 Da, ox, in green). **c** HPAEC-PAD elution patterns and MALDI-TOF MS spectra of KGM after incubation with *Mc*AA9A. The insert in panel **c** shows the putative structure of KGM: glucose (blue circles), mannose (green circles) and acetyl groups (yellow triangles). Enzymes (1 μM) were incubated in 50 mM BisTris buffer, pH 6.1, with 0.3% (w/v) KGM for 4h at 40°C with (+AscA, black spectra) or without (no AscA, red spectra) 1 mM ascorbic acid. In the presence of ascorbic acid, the HPAEC-PAD chromatogram shows stronger signals between 45 and 60 min than without ascorbic acid, which probably correspond to KGM degradation products. On MALDI-TOF, in the presence of ascorbic acid, *Mc*AA9A showed signals corresponding to native and oxidized hexose (Hex; glucose or mannose) oligomers (DP6 to DP12) with or without acetylation (Ac). Labelled peaks show the masses of monosodium adducts of native oligosaccharides (n, in black), C1-oxidized lactone or C4-oxidized ketoaldose species (Δ*m/z* -2 Da, ox, in blue) and hydrated C1-oxidized aldonic acid or C4-oxidized gemdiol species (Δ*m/z* +16 Da, ox, in green).
